# Supplementary material for: Mycobacterium tuberculosis infection is associated with increased B cell responses to unrelated pathogens
Source: Sci Rep. 2020 Aug 31;10:14324. doi: 10.1038/s41598-020-71044-4 (PMC7458924; doi:10.1038/s41598-020-71044-4)
Supplement: Supplementary file 2 — Supplementary information 2. [file 41598_2020_71044_MOESM2_ESM.docx]

*Mycobacterium tuberculosis* Infection is Associated with Increased B Cell Responses to Unrelated Pathogens

Simon G. Kimuda^1,2^, simon.kimuda@lshtm.ac.uk

Irene Andia-Biraro^1,3^, andiaodanga@yahoo.com

Ismail Sebina^#,1^, lukeismailmat@yahoo.com

Moses Egesa^1,2^, Moses.Egesa@mrcuganda.org

Angela Nalwoga^1,4^, Angela.Nalwoga@mrcuganda.org

Steven G. Smith^5^, steven.smith@lshtm.ac.uk

Bernard S. Bagaya^6^, bagayabs@yahoo.com

Jonathan Levin^7^, Jonathan.Levin@wits.ac.za

Alison M. Elliott^1,4^, [alison.elliott@lshtm.ac.uk](mailto:alison.elliott@lshtm.ac.uk)

John G. Raynes^5^, john.raynes@lshtm.ac.uk

Stephen Cose^1,2,4*^, stephen.cose@lshtm.ac.uk

1. Immunomodulation and Vaccines Programme, MRC/UVRI and LSHTM Uganda Research Unit, Entebbe, Uganda
2. Department of Medical Microbiology, School of Biomedical Sciences, Makerere University College of Health Sciences, Kampala, Uganda
3. Department of Internal Medicine, School of Medicine, Makerere University College of Health Sciences, Kampala, Uganda
4. Department of Clinical Research, London School of Hygiene & Tropical Medicine, London, United Kingdom
5. Department of Infection Biology, London School of Hygiene & Tropical Medicine, London, United Kingdom
6. Department of Immunology and Molecular Biology, School of Biomedical Sciences, Makerere University College of Health Sciences, Kampala, Uganda
7. Division of Epidemiology and Biostatistics, School of Public Health, University of the Witwatersrand, Johannesburg, South Africa

# Present address: QIMR Berghofer Medical Research Institute, Brisbane, Australia

***** Corresponding Author

Keywords: Antibodies, *Mycobacterium tuberculosis*, active pulmonary tuberculosis, latent tuberculosis infection, non-specific responses, avidity, B cells, polyclonal activation.

**
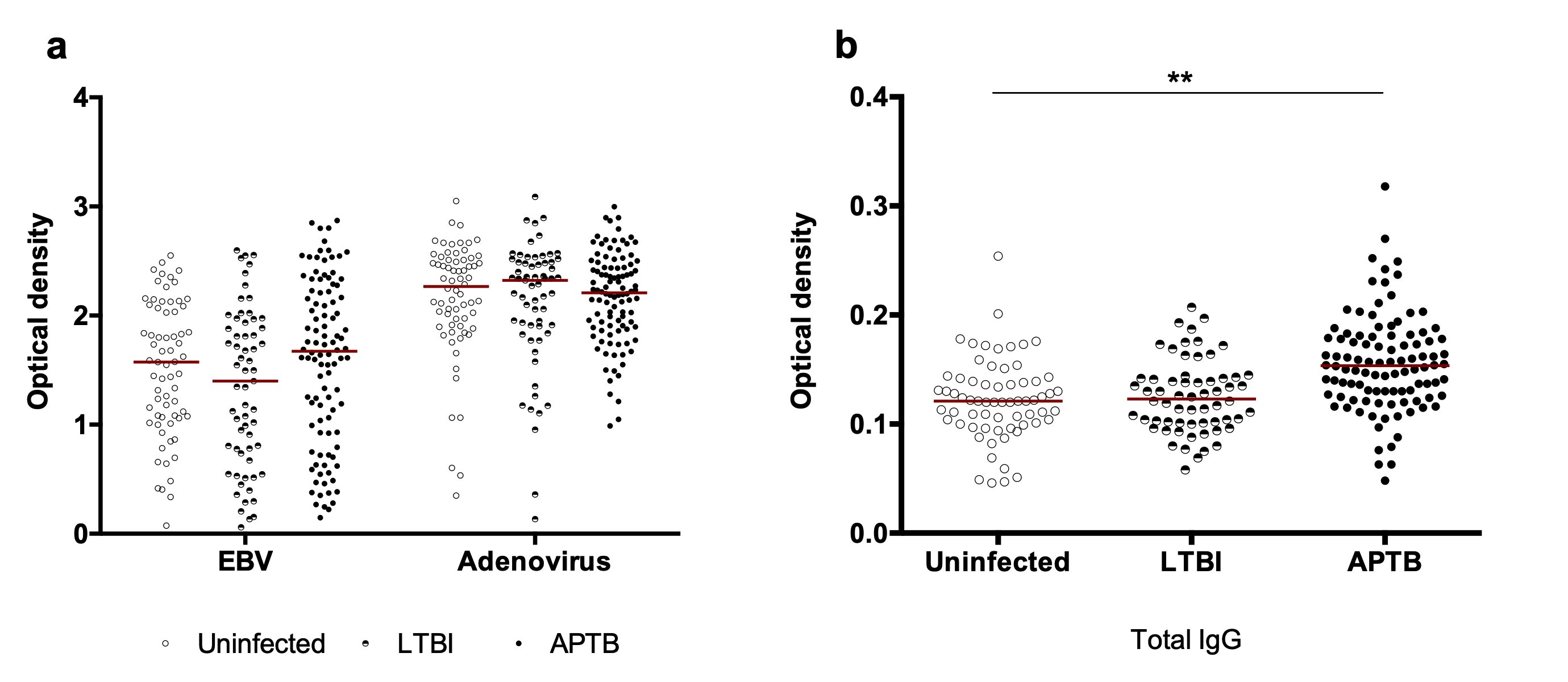
 Supplementary Figure S1: Antibody responses to EBV and adenovirus and total IgG levels in individuals of varied *M.tb* infection status.** The horizontal bars shown are median IgG antibody optical densities in each group. Antibody responses against EBV and adenovirus (panel A) at 1/100 sample dilution and total IgG levels (panel B) at a 1/100,000 sample dilution were compared across uninfected controls (n=68), individuals with LTBI (n=62) and APTB cases (n=107). The p values shown correspond to results from Kruskal–Wallis test (*p<0.05, **p<0.01). EBV: Epstein Barr virus.


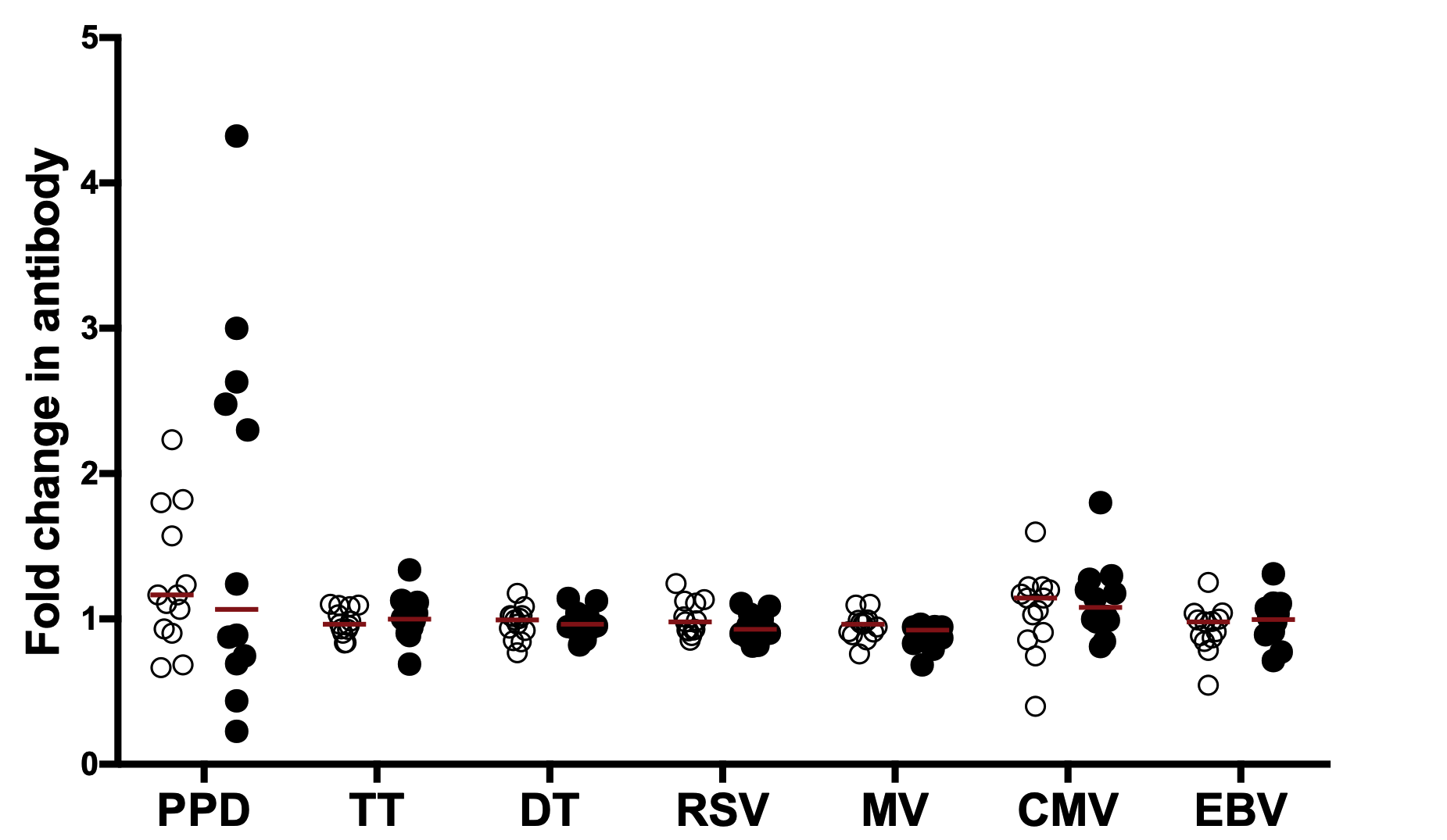


Supplementary Figure S2: Fold change in antibody responses to heterologous pathogens in BCG vaccinated individuals and their age-matched BCG naïve controls. The fold change is the ratio of antibody optical density (OD) before the time of BCG vaccination to the OD 3 weeks after BCG vaccination. The horizontal bars shown are median IgG fold changes in the antibodies in each group. The p values shown correspond to results from Wilcoxon rank sum test (*p<0.05, **p<0.01) from comparing the fold change in antibody responses in BCG vaccinated (n=12) and BCG naïve controls (n=13). PPD: purified protein derivative, TT: tetanus toxoid, DT: diphtheria toxoid, RSV: respiratory syncytial virus, MV: measles virus, CMV: cytomegalovirus, EBV: Epstein Barr virus.

**
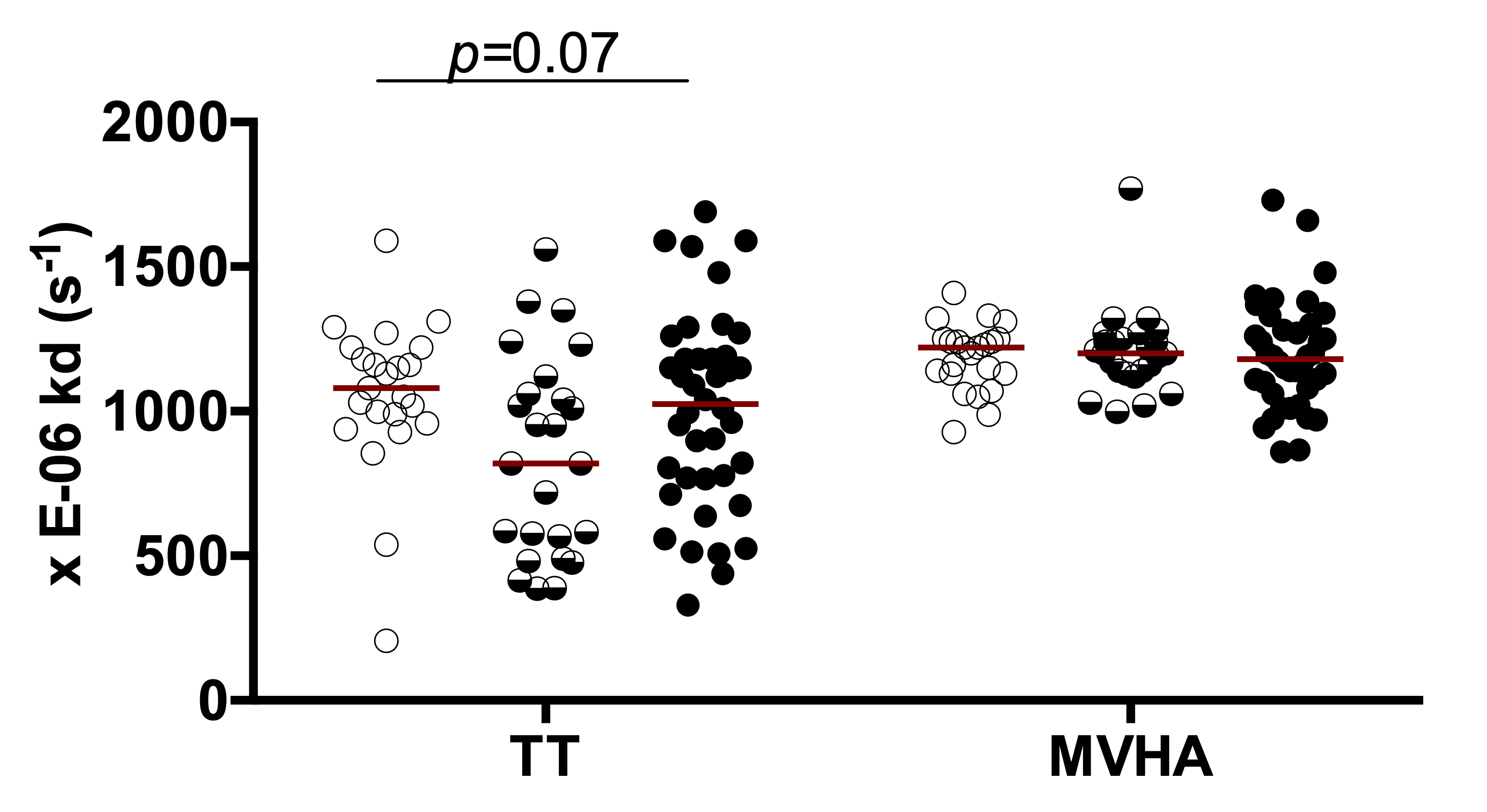
**

**Supplementary Figure S3. Variation in tetanus toxoid and measles virus-specific antibody avidity across *M.tb* infection state.** The Kruskal Wallis test was used to compare antibody dissociation rates across uninfected controls (n=23), individuals with LTBI (n=25) and APTB cases (n=40). TT: tetanus toxoid, MVHA: measles virus haemagglutinin

**Supplementary Table S1: Associations between *Mycobacterium tuberculosis* infection status and IgG antibody responses against EBV and adenovirus antigens and total IgG** *^*^*

| **Antibody optical density** | **Adjusted GMR (95%CI) ^†^** | ***p* value** | ***q* value ^ƒ^** |
| --- | --- | --- | --- |
| **Anti-EBV** |  |  |  |
| **Uninfected** | 1 |  |  |
| **LTBI** | 0.802 (0.615 - 1.045) | 0.102 | 1.000 |
| **APTB** | 0.940 (0.727 - 1.216) | 0.638 | 1.000 |
| **Anti-adenovirus** |  |  |  |
| **Uninfected** | 1 |  |  |
| **LTBI** | 0.928 (0.778 - 1.107) | 0.408 | 1.000 |
| **APTB** | 0.985 (0.892 - 1.089) | 0.769 | 1.000 |
| **Total IgG** |  |  |  |
| **Uninfected** | 1 |  |  |
| **LTBI** | 1.027 (0.926 - 1.140) | 0.610 | 1.000 |
| APTB | 1.166 (1.038 - 1.309) | 0.010 | 0.220 |

GMR: geometric mean ratio, LTBI: latent tuberculosis infection, APTB: active pulmonary tuberculosis, PPD: purified protein derivative, TT: tetanus toxoid, DT: diphtheria toxoid, RSV: respiratory syncytial virus, MV: measles virus, KSHV: Kaposi’s sarcoma herpesvirus, CMV: cytomegalovirus, EBV: Epstein Barr virus.

^*^ Linear regression analysis of antibody data from 67 uninfected controls, 62 individuals with LTBI and 89 APTB cases.

**^†^** Adjusted for age, gender, socioeconomic status and HIV infection status.

^ƒ^ q values were calculated using the Bonferroni method for comparisons between *M.tb* infection states and uninfected individuals

**Supplementary Table S2: Associations between *Mycobacterium tuberculosis* infection status and concentrations of IgG antibodies to heterologous pathogen antigens and total IgG in HIV negative individuals***^*^*

| **Antibody optical density** | **Adjusted GMR (95%CI) ^†^** | ***p value*** | ***q value ^ƒ^*** |
| --- | --- | --- | --- |
| **Anti-PPD** |  |  |  |
| **Uninfected** | 1 |  |  |
| **LTBI** | 1.226 (0.927 - 1.621) | 0.153 | 1.000 |
| **APTB** | **3.278 (2.327 - 4.616)** | **<0.001** | **0.002** |
| **Anti-TT** |  |  |  |
| **Uninfected** | 1 |  |  |
| **LTBI** | 1.229 (0.826 - 1.829) | 0.309 | 1.000 |
| **APTB** | 1.382 (0.919 - 2.077) | 0.120 | 1.000 |
| **Anti-DT** |  |  |  |
| **Uninfected** | 1 |  |  |
| **LTBI** | 1.463 (0.991 - 2.159) | 0.056 | 1.000 |
| **APTB** | 1.578 (1.132 - 2.200) | 0.007 | 0.154 |
| **Anti-RSV** |  |  |  |
| **Uninfected** | 1 |  |  |
| **LTBI** | 0.899 (0.779 - 1.036) | 0.142 | 1.000 |
| **APTB** | 1.129 (1.020 - 1.250) | 0.020 | 0.440 |
| **Anti-MV** |  |  |  |
| **Uninfected** | 1 |  |  |
| **LTBI** | 1.026 (0.743 - 1.419) | 0.875 | 1.000 |
| **APTB** | **1.613 (1.261 - 2.063)** | **<0.001** | **0.002** |
| **Anti-KSHV ORF73** |  |  |  |
| **Uninfected** | 1 |  |  |
| **LTBI** | 1.019 (0.891 - 1.165) | 0.786 | 1.000 |
| **APTB** | 1.103 (0.938 - 1.298) | 0.237 | 1.000 |
| **Anti-KSHV K8.1** |  |  |  |
| **Uninfected** | 1 |  |  |
| **LTBI** | 1.057 (0.631 - 1.771) | 0.832 | 1.000 |
| **APTB** | 1.887 (1.066 - 3.341) | 0.029 | 0.638 |
| **Anti-CMV** |  |  |  |
| **Uninfected** | 1 |  |  |
| **LTBI** | 0.925 (0.614 - 1.393) | 0.707 | 1.000 |
| **APTB** | 1.075 (0.794 - 1.455) | 0.642 | 1.000 |
| **Anti-EBV** |  |  |  |
| **Uninfected** | 1 |  |  |
| **LTBI** | 0.746 (0.570 - 0.978) | 0.034 | 0.748 |
| **APTB** | 0.87 (0.664 - 1.140) | 0.312 | 1.000 |
| **Anti-adenovirus** |  |  |  |
| **Uninfected** | 1 |  |  |
| **LTBI** | 0.906 (0.761 - 1.079) | 0.27 | 1.000 |
| **APTB** | 0.962 (0.865 - 1.069) | 0.471 | 1.000 |
| **Total IgG** |  |  |  |
| **Uninfected** | 1 |  |  |
| **LTBI** | 1.035 (0.925 - 1.157) | 0.551 | 1.000 |
| **APTB** | 1.197 (1.049 - 1.367) | 0.008 | 0.176 |

GMR: geometric mean ratio, LTBI: latent tuberculosis infection, APTB: active pulmonary tuberculosis, PPD: purified protein derivative, TT: tetanus toxoid, DT: diphtheria toxoid, RSV: respiratory syncytial virus, MV: measles virus, KSHV: Kaposi’s sarcoma herpesvirus, CMV: cytomegalovirus, EBV: Epstein Barr virus.

^*^ Linear regression analysis of antibody data from 63 uninfected controls, 57 individuals with LTBI and 55 APTB cases.

**^†^** Adjusted for age, gender and socioeconomic status

^ƒ^ q values were calculated using the Bonferroni method for comparisons between *M.tb* infection states and uninfected individuals

**Supplementary Table S3: Association between *M.tb* infection state and SPR derived TT and MVHA specific antibody dissociation rates in HIV negative individuals ^‡^**

| **Dissociation rate E-06 [kd(s-1)]** | **Adjusted GMR (95%CI) ^†^** | ***p value*** | ***q value ^ƒ^*** |
| --- | --- | --- | --- |
| **Anti-TT** |  |  |  |
| **Uninfected** | 1 |  |  |
| **LTBI** | 0.759 (0.562 - 1.025) | 0.072 | 0.288 |
| **APTB** | **0.71 (0.555 - 0.908)** | **0.006** | **0.024** |
| **Anti-MVHA** |  |  |  |
| **Uninfected** | 1 |  |  |
| **LTBI** | 0.977 (0.913 - 1.046) | 0.511 | 1.000 |
| **APTB** | 0.919 (0.845 - 1.000) | 0.049 | 0.196 |

GMR: geometric mean ratio, LTBI: latent tuberculosis infection, APTB: active pulmonary tuberculosis, TT: tetanus toxoid, MVHA: measles virus haemagglutinin antigen

^‡^ 21 uninfected controls, 23 individuals with LTBI and 22 APTB cases

^¥^ Adjusted for age, gender and socioeconomic status

**^∫^** q values were calculated using the Bonferroni method for comparisons between *M.tb* infection states and uninfected individuals
